# Assessment of Total Phenolic, Antioxidant, and Antibacterial Activities of *Passiflora* Species

**DOI:** 10.1155/2014/167309

**Published:** 2014-01-21

**Authors:** Shiamala Devi Ramaiya, Japar Sidik Bujang, Muta Harah Zakaria

**Affiliations:** ^1^Department of Animal Science and Fisheries, Faculty of Agriculture and Food Sciences, Universiti Putra Malaysia Bintulu Sarawak Campus, 97008 Bintulu, Sarawak, Malaysia; ^2^Department of Aquaculture, Faculty of Agriculture, Universiti Putra Malaysia (UPM), 43400 Serdang, Selangor Darul Ehsan, Malaysia

## Abstract

This study focused on total phenolic content (TPC) and antioxidant and antibacterial activities of the leaves and stems of *Passiflora quadrangularis*, *P. maliformis*, and *P. edulis *extracted using three solvents: petroleum ether, acetone, and methanol. The maximum extraction yields of antioxidant components from the leaves and stems were isolated using methanol extracts of *P. edulis *(24.28%) and *P. quadrangularis *(9.76%), respectively. Among the leaf extracts, the methanol extract of *P. maliformis *had the significantly highest TPC and the strongest antioxidant activity, whereas among the stem extracts, the methanol extract of *P. quadrangularis *showed the highest phenolic amount and possessed the strongest antioxidant activity. The antibacterial properties of the *Passiflora *species were tested using the disc diffusion method against 10 human pathogenic bacteria. The largest inhibition zone was observed for the methanol extract of *P. maliformis *against *B. subtilis*. Generally, extracts from the *Passiflora *species exhibit distinct inhibition against Gram-positive but not Gram-negative bacteria. Based on the generated biplot, three clusters of bacteria were designated according to their performance towards the tested extracts. The present study revealed that methanol extracts of the *Passiflora* contain constituents with significant phenolic, antioxidant, and antibacterial properties for pharmaceutical and nutraceutical uses.

## 1. Introduction

Natural products have received significant interest as source for new drug development in biomedical research. The modern pharmaceutical industry is highly dependent on plant-based medicines, with more than 50% of drug substances derived from natural resources [1]. Plants are known to produce phytochemicals, which are potential sources of anticarcinogenic, anticancer, antimicrobial, and antioxidant activity; these compounds include flavonoids, phenolic acids, and tannins [2, 3]. Research has focused on the discovery of clinically useful antimicrobial drugs and functional food from natural resources for pharmaceutical and nutraceutical uses [4, 5]. Additionally, the increasing interest in traditional ethnomedicine may lead to the discovery of novel therapeutic agents.

Passion fruit is an agronomically important crop and is used commercially in the fruit industry. This plant belongs to the genus *Passiflora *and is extensively grown in the tropical and subtropical regions of the world. Passion fruit is widely distributed over Central America and South America, with more production in the latter region [6]. Brazil is the major producer and consumer of passion fruit worldwide. In Malaysia, this fruit is cultivated on a small scale due to the prevalence of suitable growing conditions and increase in demand [7].

In recent years, researchers have shown increasing interest in the passion fruit plant due to its phytotherapeutic properties, ethnobotanical uses, chemotaxonomic information, and the interaction of the plant with its environment; these factors have been suggested as selection criteria for potential sources of natural molecules of pharmaceutical relevance [8]. The leaves, stems, roots, and fruits of *Passiflora *species have long been used in folk medicine and are finding an increasingly important place in modern medicine. Traditionally, the flower of *Passiflora* has been valued medicinally for its sedative, antispasmodic, anxiolytic, and hypotensive activity, as well as its sleep-inducing effects [6, 9–11]. Although a number of species, including *P. edulis* and *P. quadrangularis*, have been valued for the above purposes, *P. incarnata* has demonstrated the strongest effects, and its efficacy is comparable with that of other species [12–16]. The ethnobotanical literature has also indicated that the *Passiflora *plant contains a variety of compounds, including alkaloids, phenols, glycoside flavonoids, and cyanogenic constituents [10]. The leaf extract of *Passiflora *species has been shown to possess anxiolytic and sedative activity [17–19], as well as treatment for diabetes and hypertension [20], and anti-inflammatory [21], cytotoxic [22], antioxidant [23, 24], antibacterial [25–27], and antifungal properties [28]. In support of these claims, a study by Birner and Nicolls [29] has reported the isolation of an antibacterial and antifungal compound called Passicol from *P. edulis*. This plant has a continuing history of use in Ayurveda and homeopathic medicine as a treatment for a number of ailments [30]. The seeds possess an antifungal protein (Passiflin) [31] and an antifungal peptide (Pe-AFP1), which protect the plant from invasion by pathogenic fungi [32].

From the above review, it may be concluded that although many studies have examined the ethnobotanical attributes and medicinal uses of purple passion fruits, little information exists on phenolic content, antioxidant capacities, and antibacterial properties of other *Passiflora* species. To fill this gap, the present study screens various parts of several *Passiflora* species for the total phenolic content and antioxidant and antibacterial activity extracted using three different solvents: petroleum ether, acetone, and methanol.

## 2. Materials and Methods

### 2.1. Plant Materials

Leaves and stems of *P. quadrangularis, P. maliformis*, and* P. edulis* were collected randomly from 10 plants of each species at the passion fruit farm at Universiti Putra Malaysia Bintulu Sarawak Campus (UPMKB), Bintulu, Sarawak. Specimen identification and botanical nomenclature were based on the scheme of Ulmer and MacDougal [6]. The plant parts were brought to the laboratory and immediately inspected, cleaned with distilled water, and dried in the shade at room temperature for 2 weeks. The dried parts were homogenized to a fine powder and stored in airtight containers until used for the analyses described below.

### 2.2. Preparations of Extracts

For each of the dried parts, 5 g of powered sample was separately extracted with 50 mL of three different solvents (petroleum ether, acetone, and methanol) using a shaking water bath at 80 rpm for 48 hours at room temperature. The extracts were then centrifuged at 500 ×g for 10 min and filtered through Whatman No. 2 filter paper. Each extract was then evaporated to dryness. The concentrated extracts were suspended in dimethyl sulfoxide (DMSO) and stored in a refrigerator at 4°C prior to the analyses.

### 2.3. Determination of Total Phenolic Content (TPC)

TPC was determined using the Folin-Ciocalteu (Merck) method as described by Asami et al. [33]. The extract solution in appropriate solvent (1 mL) was added with 0.3 mL of Folin-Ciocalteu reagent. Six minutes later, 10 mL of 7% sodium carbonate solution was added, mixed well, and left it for 2 hours. The absorbance readings were taken at 740 nm on an 1100 Series spectrophotometer. The experiment was performed in triplicate. The quantification of TPC was conducted using a calibration curve prepared with a gallic acid standard (*R* ^2^ = 0.997). The results were expressed as g garlic acid equivalent (GAE) per 100 g DW of extract.

### 2.4. 2,2-Diphenyl-1-picrylhydrazyl (DPPH) Radical Scavenging Assay

Total antioxidant activity (TAA) of the *Passiflora* extracts against DPPH radicals was determined according to the modified methods of Brand-Williams et al. [34]. Three milliliter DPPH (100 *μ*M) in methanol was added to 1.0 mL of *Passiflora *extract. After 30 min incubation period at room temperature the absorbance was taken against blank prepared without extract at 517 nm on an 1100 Series spectrophotometer. The concentration of sample required to scavenge 50% DPPH (EC_50_) was determined by linear regression for the concentration and EC_50_ (%). The experiment was performed in triplicate, and the results were expressed as *μ*g/mL^−1^. A lower EC_50_ value indicates a higher antioxidant activity.

### 2.5. Bacterial Strains

The plant extracts were individually tested against 10 human pathogenic bacteria: the 5 Gram*-*positive bacteria* Bacillus subtilis *(ATCC 6633),* Bacillus cereus *(ATCC 11778), *Listeria monocytogenes* (ATCC 7644), *Streptococcus gallolyticus* (ATCC 49147), and *Staphylococcus aureus* (MTCC 554231) and the five Gram-negative bacteria *Pseudomonas aeruginosa *(ATCC 27853), *Klebsiella oxytoca* (ATCC 49131), *Proteus vulgaris* (ATCC 49132), *Salmonella enteritidis* (MTCC 125239), and *Escherichia coli *(MTCC 423155). The bacterial strains were obtained from Thermo Fisher Scientific. The bacterial strains were cultured overnight at 37°C in nutrient broth. The cultures were then maintained at 4°C and were subcultured prior to analysis.

### 2.6. Antibacterial Activity

The antibacterial activity of the *Passiflora* species extracts was studied by the disc diffusion method as reported by Lalitha [35]. The turbidity of each bacterial suspension was adjusted to a 0.5 McFarland standard, with each suspension containing 1.5 × 10^8^ CFU/mL. The bacterial strains were spread individually in sterile Petri dishes on prepared nutrient agar medium. Sterilized filter paper discs (5.5 mm in diameter) were impregnated with 5 *μ*L of 50 *μ*g/*μ*L extract (250 *μ*g/disc) and placed on the surface of the agar plates that had previously been inoculated with the tested bacteria. A disc impregnated with chloramphenicol (10 *μ*g/disc) was used as a standard, while the respective solvents were used as the negative controls. The agar plates were incubated at 37°C for 24 hours. The antibacterial activity was examined by measuring the diameters of the growth inhibition zones (mm) for the tested pathogenic bacteria compared to the standards. The measurement of the inhibition zones was conducted using three sample replications.

### 2.7. Statistical Analysis

The statistical software SAS 9.0 was used for data analysis. Means were compared using single factor analysis of variance (ANOVA). A post hoc Tukey's test (*P* < 0.05) was performed if the ANOVA result was significant. Principal component analysis (PCA) based on the Pearson method was conducted using XLSTAT software to determine the relationship between the activity of the plant parts extracts and the pathogenic bacteria.

## 3. Results and Discussion 

### 3.1. Extract Yields

The extract yields of the leaves and stems of *P. quadrangularis, P. maliformis,* and *P. edulis* obtained using the three extraction solvents are presented in Table 1. The extraction yields showed significant differences among the *Passiflora* species and the different solvents tested. The extract yields of the leaves and stems ranged from 3.70 to 24.28% and 1.53 to 9.76% (per 5 g dry weight), respectively. Methanol was the most effective extractant of antioxidant compounds, followed by acetone and petroleum ether. This trend was similar to the findings of Gahlaut and Chhillar [36]. Both methanol and ethanol have been established as effective solvents for extracting antioxidant compounds from plant materials [37]. Methanol yielded the greatest percentage of crude extract from the leaves of *P. edulis* (24.28 ± 0.67%), whereas lower yields were obtained from the petroleum ether extracts of *P. edulis *and* P. maliformis*, 4.39 ± 0.46% and 3.70 ± 0.97%, respectively. For the stem, the highest percentage of crude extract was obtained from the methanol extracts of *P. quadrangularis* (9.76 ± 0.20%), and the lowest extract yield was recorded from the petroleum ether extracts of *P. maliformis *(1.53 ± 0.11%). The present study revealed that the extraction yield varied with the solvents used and the chemical properties of the extractable components in each plant part [5].

### 3.2. Total Phenol Content (TPC)

The total phenolic content varied between the different plant parts of the *Passiflora* species with respect to the extraction solvent used (petroleum ether, acetone, or methanol). The phenolic content for the extracted leaves and stems ranged from 3.32 to 1.23 g GAE/100 g and 3.74 to 1.03 g GAE/100 g, respectively (Table 1). Among the three solvents, methanol recovered the maximum TPC from the leaves and stems. Petroleum ether was least effective at extracting phenolic compounds. Among the leaf extracts, the methanol extract from *P. maliformis* showed the highest phenolic content (3.32 ± 0.06 g GAE/100 g), followed by the methanol extract of *P. edulis* at 2.37 ± 0.11 g GAE/100 g, whereas petroleum ether and acetone extracts from *P. quadrangularis* produced had lower phenolic contents. In a comparison of the results obtained for the TPC of the *P. edulis* leaves with those reported in the literature, similar values have been reported for* P. alata*: 3.42 ± 0.39 g GAE/100 g for ethanol and 1.40 ± 0.49 g GAE/100 g for acetone extracts [38]. However, the present TPC values for the leaves of *P. edulis* were higher than those obtained by Silva et al. [39] (0.83 ± 0.07 g GAE/100 g) and four times lower than those reported by Rudnicki et al. [23] (9.25 g GAE/100 g).

The stem extracts of *P. quadrangularis* had the highest TPC value among all of the extracts. Phenolic compounds are widely distributed in plants and have garnered attention due to their antimutagenic, antitumor, and antioxidant properties, which contribute to human health [40]. The variation in TPC values may be attributed to the plant origins of the extractable compounds and the efficacy of the solvents used to recover the polyphenols from the plant materials. Similar variations have been described for *Passiflora* [23, 41] and for other plants, for example, *Pongamia pinnata* [5] and *Hippophae salicifolia* [42]. The variation could also be influenced by geographical origin, cultivar, harvesting, and drying method [43].

### 3.3. Total Antioxidant Activity (TAA)

Significant variation of TAA was observed among the different extracts of the* Passiflora* species and is presented in Table 1. The present results indicate that the extracts exhibited potential free radical scavenging activity. A lower EC_50_ value indicates a greater antioxidant activity for a given extract. The TAA ranged from 456.9 to 3423.8 *μ*g/mL and 313.7 to 2137.2 *μ*g/mL for the leaves and stems, respectively. Among the extracts, the strongest TAA was observed in the methanol, followed by the acetone, with the lowest antioxidant activity observed in the petroleum ether extracts. In accordance with the TPC results, the antioxidant activity of the leaf was observed to be highest in the methanol extract of *P. maliformis* (456.9 ± 13.1 *μ*g/mL), followed by those of *P. edulis* (653.5 ± 6.1 *μ*g/mL) and *P. quadrangularis *(785.2 ± 1.8 *μ*g/mL). The TAA values obtained in this study agreed with the findings of  Vasic et al. [38] for the methanol and acetone extracts of* P. alata, *808.69 *μ*g/mL and 1107.79 *μ*g/mL, respectively. Similarly, the TAA value of the *P. edulis *leaf extract was comparable to that recorded by Sunitha and Devaki [24] (875 *μ*g/mL) using ethanol extracts. The present TAA value for *P. edulis* was higher than that reported previously by Silva et al. [39] (1100 *μ*g/mL) for methanol leaf extract but lower than those reported by Ripa et al. [22] (58.88 *μ*g/mL, using petroleum ether).

The strongest antioxidant activity in the stem was recorded from the methanol extracts for *P. quadrangularis *(313.7 ± 1.2 *μ*g/mL), followed by those of *P. edulis *(429.6 ± 3.6 *μ*g/mL) and* P. maliformis *(973.0 ± 3.7 *μ*g/mL). The petroleum ether extract of the *P. maliformis* stem was found to possess the weakest activity (2137.2 ± 2.7 *μ*g/mL). The TAA values of the stem extracts in the present study were lower than that of the petroleum ether extracts (54.01 *μ*g/mL) of* P. edulis* as reported by Ripa et al. [22]. In general, a higher TPC value led to stronger antioxidant activity. Several authors have mentioned this relationship in previous studies, for example, those of *Passiflora* species [44] and* Veronica *species [45]. TPC could be considered as an important indicator of the antioxidant properties of plant extracts. Although TPC has a strong correlation with antioxidant activity, other constituents, such as flavonoids, alkaloids, glycosides, carotenoids, vitamins, and other secondary metabolites, may also be contributing factors [46].

### 3.4. Antibacterial Activities

The antibacterial activities of the leaves and stems extract of the *Passiflora *species were tested against 10 human pathogenic bacteria; the results of the tests on the 5 Gram-positive and 5 Gram-negative bacteria are presented in Tables 2 and 3. The observed antibacterial activities were categorized as follows: (a) sensitive-inhibition zone, >18 mm; (b) intermediate-inhibition zone, 13–17 mm; and (c) resistance-inhibition zone, <13 mm [47]. The methanol extracts exhibited considerable antibacterial activity against the bacteria tested. The activity of the methanol extracts might be partly due to their higher phenolic and antioxidant contents. The largest inhibition zone was produced by the methanol leaf extract of *P. maliformis* against *B. subtilis* (22.5 ± 0.8 mm, sensitive zone). *Bacillus cereus* and *S. gallolyticus *were also sensitive to the methanol leaf extract of *P. maliformis *(inhibition zones of 18.7 ± 0.2 mm and 20.5 ± 0.5 mm, resp.). These results showed that the methanol extracts have considerable antibacterial potency despite their crude form.

**Figure 1:**
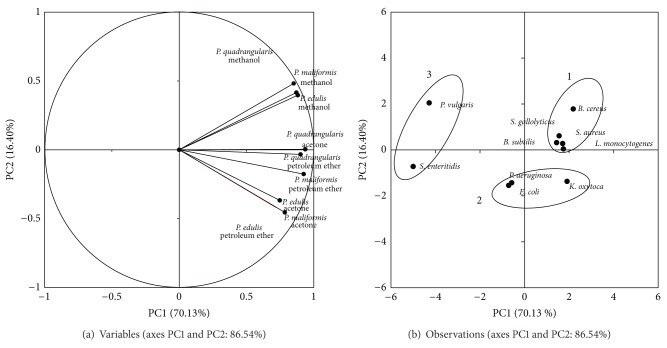
(a) Plot of the variables tested against pathogenic microbes for leaves extracts. Percentages in parentheses represent the variation of each component. (b) Positions of the PC scores of the 10 microorganisms according to PC1 and PC2.

**Figure 2:**
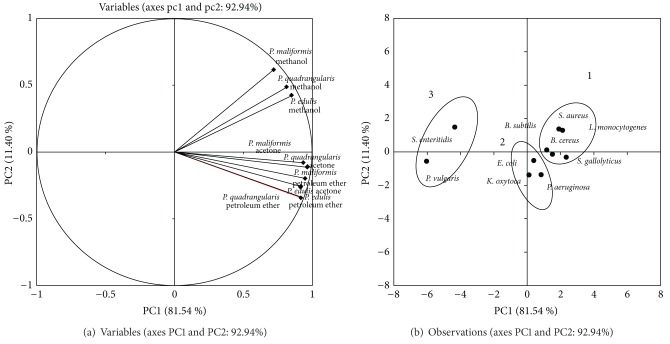
(a) Plot of the variables tested against pathogenic microbes for stems extracts. Percentages in parentheses represent the variation of each component. (b) Positions of the PC scores of the 10 microorganisms according to PC1 and PC2.

The potential antibacterial activities of the extracts against the 10 human pathogens are analyzed using PCA and illustrated as biplots in Figures 1 (leaf) and 2 (stem). The PCA indicates that the first two PCs for the leaf extracts accounted for 86.54% of the total variance. PC1 explained a higher percentage of total variance (70.13%) than did PC2 (16.40%). For the stem extracts, the first two principal components explained 92.94% of the total variance, with PC1 and PC2 representing 81.54% and 11.40% of the total variance, respectively. The methanol extracts tested were loaded heavily on the positive sites of PC1 and PC2, while the acetone and petroleum ether extracts were connected to the positive sites of PC1 and negative sites of PC2 in both the leaf and stem biplots (Figures 1(a) and 2(a)). The 10 pathogens examined clustered into three main groups in both leaf and stem biplots. For the biplot of the leaf extracts (Figure 1(b)), the first group consisted of *L. monocytogenes, S. gallolyticus, S. aureus, B. subtilis*, and *B. cereus*. This group represents the Gram-positive bacteria, and all the extracts from the three tested solvents showed significant antibacterial activities against this group. The methanol leaf extracts of the *Passiflora* species exhibited intermediate activity against *S. aureus *(14.5 ± 0.6 mm), *B. subtilis* (14.0 ± 0.6 mm), and *L. monocytogenes* (14.5 ± 0.5 mm), whereas the petroleum ether and acetone extracts showed smaller zones of inhibition against the other tested pathogenic bacteria. Moderate inhibition was also observed from the methanol and acetone extracts of *P. quadrangularis* against *S. aureus* and *B. cereus. Staphylococcus aureus* was also moderately susceptible (14.2 ± 0.6 mm) to the methanol extract of *P. maliformis*. The acetone and petroleum ether extracts of *P. maliformis* showed intermediate-inhibition zones against *S. gallolyticus *(16.3 ± 0.3 and 13.5 ± 0.1 mm, resp.).

The second group was composed of *P. aeruginosa*, *E. coli*, and *K. oxytoca *which are categorized as Gram-negative bacteria and exhibited some degree of sensitivity towards all the extracts.The leaf and stem extracts, as well as the standard, showed weak antibacterial activities towards both *P. aeruginosa* and* E. coli*. *Klebsiella oxytoca* was highly sensitive (21.4 ± 0.8 mm) to the acetone extracts and moderately susceptible (~14.0 mm) to the methanol and petroleum ether extracts of *P. maliformis*. The third group consisted of *P. vulgaris* and *S. enteritidis*, and these bacteria were sensitive to the methanol extracts of the *Passiflora *leaves. Different species exhibited varying degrees of sensitivity to the antibacterial activity of the extracts. These differences can be attributed to the presence of natural antimicrobial compounds in the different parts and species of the *Passiflora* plants. The antibacterial activity of the *P. edulis *leaf extract against *S. aureus* in the present study was similar to the levels reported by Akanbi et al. [27] (12.0 mm) and Kannan et al. [48] (10 ± 1.03 mm) for methanol extracts. Johnson et al. [49] reported that chloroform and methanol extracts of the callus tissue and leaves of *P. edulis* possessed potential antimicrobial activity against *S. aureus*. The antibacterial inhibition against *B. subtilis* in the present results was slightly lower than that obtained (18.0 ± 0.88 mm) by Kannan et al. [48].

Similarly, for the stem extracts (Figure 2(b)), the first group consisted of the Gram-positive bacteria: *L. monocytogenes, S. gallolyticus, S. aureus, B. subtilis*, and *B. cereus*. The methanol and acetone extracts of *P. maliformis* and the methanol extracts of *P. quadrangularis* exhibited intermediate inhibition against *S. aureus, S. gallolyticus*, and *B. subtilis*. The second group comprised the Gram-negative bacteria: *P. aeruginosa, K. oxytoca*, and* E. coli*. The *S. enteritidis*, which was resistant to only the methanol extracts, and the *P. vulgaris* that was not inhibited by the extracts were clustered in the last group. The obtained values for the antibacterial activities of the stem extracts against *S. aureus, B. subtilis, P. aeruginosa*, and* E. coli *were within the range of previous studies of *P. edulis* stem extracts [22, 27]. The PCA showed significant variation between the Gram-positive and Gram-negative bacteria. This result was in agreement with the fact that Gram-negative bacteria possess a unique outer membrane of lipopolysaccharide, which protects them from the permeation of active compounds [50]. The tested extracts showed potential activity against the Gram-positive bacteria; *L*. *monocytogenes*, *S. gallolyticus*, *S. aureus, B. subtilis*, and* B. cereus* were all susceptible to the *Passiflora* extracts, which may be attributed to the presence of a single membrane that makes these bacteria more accessible to the penetration of active plant compounds [51]. This work provides insight into the therapeutic properties of *Passiflora* in traditional medicine. Further research is required to study the isolates of this plant's bioactive compounds and to evaluate the mechanisms of action for their antioxidant and antibacterial activities.

## 4. Conclusions

The results confirmed the ethnobotanical views of the *Passiflora* species, which are used in traditional medicine to treat the various infectious diseases caused by the microbes. Methanol was established to be the most effective among the tested solvents at recovering the phenolic and antioxidant contents from the different parts of the *Passiflora* species. The methanol extract also contained certain constituents with significant antibacterial properties. Gram-negative bacteria were generally less susceptible to the *Passiflora* extracts than were Gram-positive bacteria. This contrast was illustrated in the biplots generated from the PCA. Although the materials employed in this study are generally considered as plant wastes, they can be used as sources of bioactive constituents. The present study establishes that the leaves and stems of the *Passiflora *species could be utilized for treating ailments, giving the plants value beyond that of their fruits, which are processed as juice and other products.

## Figures and Tables

**Table 1:** Yields, TPC, and TAA of different solvent extracts of *Passiflora *species.

Variable	Solvent extracts of *Passiflora *species								
	*Passiflora edulis *			*Passiflora quadrangularis *			*Passiflora maliformis *		
	PE	ACT	MET	PE	ACT	MET	PE	ACT	MET
	Leaves								
Yield (%)	4.39 ± 0.46^f^	10.15 ± 0.03^cd^	24.28 ± 0.67^a^	7.73 ± 0.53^e^	12.14 ± 0.72^c^	16.73 ± 0.78^b^	3.70 ± 0.97^f^	9.21 ± 0.59^de^	15.56 ± 0.56^b^
TPC (g GAE/100 g)	1.98 ± 0.07^bc^	2.03 ± 0.08^bc^	2.37 ± 0.11^b^	1.23 ± 0.05^d^	1.39 ± 0.06^d^	2.17 ± 0.43^b^	1.52 ± 0.36^cd^	1.71 ± 0.20^bcd^	3.32 ± 0.06^a^
TAA (*µ*g/mL)	2338.0 ± 12.6^a^	1499.6 ± 11.3^bc^	653.5 ± 6.1^de^	3423.8 ± 5.5^a^	1715.2 ± 9.1^bc^	785.2 ± 1.8^de^	2113.1 ± 3.9^ab^	1264.6 ± 11.2^cd^	456.9 ± 13.1^e^

	Stems								
Yield (%)	2.58 ± 0.18^cde^	3.17 ± 0.23^c^	4.33 ± 0.38^b^	2.17 ± 0.19^ef^	3.98 ± 0.18^b^	9.76 ± 0.20^a^	1.53 ± 0.11^f^	2.40 ± 0.35^de^	3.07 ± 0.06^cd^
TPC (g GAE/100 g)	2.01 ± 0.08^c^	2.21 ± 0.16^c^	2.58 ± 0.06^b^	1.52 ± 0.04^d^	2.68 ± 0.28^b^	3.74 ± 0.24^a^	1.03 ± 0.15^e^	1.07 ± 0.14^de^	1.28 ± 0.07^de^
TAA (*µ*g/mL)	1765.1 ± 8.2^b^	1085.7 ± 8.5^c^	429.6 ± 3.6^d^	1730.4 ± 10.5^b^	1080.5 ± 9.8^c^	313.7 ± 1.2^d^	2137.2 ± 2.7^a^	1807.2 ± 3.1^b^	973.0 ± 3.7^c^

Means in the same row with a different letter (a–f) are significantly different (Tukey's test, *P* < 0.05). PE: petroleum ether; ACT: acetone; MET: methanol.

**Table 2:** Antibacterial activity of different solvent extracts from the leaves of *Passiflora* species.

Bacteria	Plant extracts inhibition zone diameter in mm									STD
	*Passiflora edulis *			*Passiflora quadrangularis *			*Passiflora maliformis *			
	PE	ACT	MET	PE	ACT	MET	PE	ACT	MET	
Gram-positive bacteria										
*S. aureus *	8.4 ± 0.5^e^	11.4 ± 0.4^cd^	14.5 ± 0.6^b^	12.3 ± 0.8^bc^	13.7 ± 0.6^bc^	13.3 ± 0.3^bc^	8.3 ± 0.4^e^	9.1 ± 0.3^de^	14.2 ± 0.6^b^	24.7 ± 0.6^a^
*B. cereus *	10.2 ± 0.2^ef^	9.8 ± 0.3^ef^	17.5 ± 0.4^b^	8.0 ± 0.2^f^	13.6 ± 0.3^cd^	15.7 ± 0.4^c^	11.9 ± 0.1^de^	10.8 ± 0.2^*def*^	18.7 ± 0.2^b^	28.6 ± 0.7^a^
*B. subtilis *	8.7 ± 0.1^e^	10.6 ± 0.6^de^	14.0 ± 0.6^c^	9.1 ± 0.3^e^	10.5 ± 0.2^de^	9.3 ± 0.2^e^	10.4 ± 0.2^de^	11.3 ± 0.2^d^	22.5 ± 0.8^b^	30.8 ± 0.8^a^
*L. monocytogenes *	10.1 ± 0.3^b^	13.2 ± 1.0^b^	14.5 ± 0.5^b^	9.6 ± 0.5^b^	10.4 ± 0.6^b^	12.5 ± 0.4^b^	12.6 ± 1.1^b^	10.8 ± 0.3^b^	14.2 ± 0.7^b^	21.7 ± 0.5^a^
*S. gallolyticus *	8.7 ± 0.4^e^	10.3 ± 0.2^de^	11.6 ± 0.2^de^	11.2 ± 0.2^de^	8.6 ± 0.2^e^	12.1 ± 0.1^cde^	13.5 ± 0.3^cd^	16.3 ± 0.1^c^	20.5 ± 0.5^b^	28.3 ± 0.2^a^
Gram-negative bacteria										
*P. aeruginosa *	9.7 ± 0.4^bc^	9.8 ± 0.2^bc^	10.6 ± 0.5^ab^	7.2 ± 0.4^de^	6.2 ± 0.2^e^	8.5 ± 0.3^cd^	7.4 ± 0.5^de^	7.7 ± 0.4^de^	8.3 ± 0.4^cd^	12.3 ± 0.6^a^
*K. oxytoca *	10.4 ± 0.8^d^	13.3 ± 0.6^c^	11.2 ± 0.4^d^	9.2 ± 0.6^d^	9.5 ± 0.1^d^	9.3 ± 0.1^d^	14.2 ± 0.9^c^	21.4 ± 0.8^b^	14.5 ± 0.3^c^	26.2 ± 0.5^a^
*P. vulgaris *	0.0 ± 0.0	0.0 ± 0.0	7.6 ± 0.3^c^	0.0 ± 0.0	0.0 ± 0.0	15.0 ± 0.6^b^	0.0 ± 0.0	0.0 ± 0.0	14.7 ± 0.4^b^	23.3 ± 0.6^a^
*S. enteritidis *	0.0 ± 0.0	0.0 ± 0.0	6.8 ± 0.5^b^	0.0 ± 0.0	0.0 ± 0.0	6.8 ± 0.2^b^	0.0 ± 0.0	0.0 ± 0.0	7.6 ± 0.3^b^	21.5 ± 0.2^a^
*E. coli *	9.1 ± 0.6^ab^	7.2 ± 0.3^b^	10.6 ± 0.3^a^	7.0 ± 0.1^b^	7.1 ± 0.5^b^	7.2 ± 0.5^b^	9.1 ± 0.1^ab^	7.0 ± 0.6^b^	9.0 ± 0.2^ab^	11.3 ± 0.1^a^

Different superscript letters within the same row indicate significant differences (Tukey's test, *P* < 0.05) of the means for each solvent extract of the *Passiflora*. PE: petroleum ether; ACT: acetone; MET: methanol; STD: standard (chloramphenicol).

**Table 3:** Antibacterial activity of different solvent extracts from the stems of *Passiflora* species.

Bacteria	Plant extracts inhibition zone diameter in mm									STD
	*Passiflora edulis *			*Passiflora quadrangularis *			*Passiflora maliformis *			
	PE	ACT	MET	PE	ACT	MET	PE	ACT	MET	
Gram-positive bacteria										
*S. aureus *	7.3 ± 0.2^e^	8.3 ± 0.2^e^	9.3 ± 0.3^de^	9.6 ± 0.6^de^	11.7 ± 0.5^cd^	16.2 ± 0.2^b^	9.0 ± 0.1^e^	13.4 ± 0.1^c^	14.7 ± 0.5^bc^	24.7 ± 0.6^a^
*B. cereus *	10.4 ± 0.2^bcd^	8.7 ± 0.2^d^	12.5 ± 0.7^b^	9.5 ± 0.4^cd^	8.2 ± 0.1^d^	10.1 ± 0.6^bcd^	8.5 ± 0.2^d^	9.6 ± 0.2^cd^	11.3 ± 0.3^bc^	28.6 ± 0.7^a^
*B. subtilis *	7.0 ± 0.1^f^	10.3 ± 0.3^d^	11.7 ± 0.5^cd^	8.5 ± 0.4^f^	10.4 ± 0.2^d^	13.3 ± 0.3^c^	10.1 ± 0.6^de^	15.1 ± 0.2^b^	15.4 ± 0.1^b^	30.8 ± 0.8^a^
*L. monocytogenes *	11.3 ± 0.3^b^	11.0 ± 0.4^b^	11.2 ± 0.8^b^	13.0 ± 0.5^b^	10.9 ± 0.5^b^	12.1 ± 1.0^b^	10.7 ± 0.8^b^	11.0 ± 0.4^b^	11.2 ± 0.9^b^	21.7 ± 0.5^a^
*S. gallolyticus *	10.0 ± 0.5^b^	9.7 ± 0.1^b^	10.4 ± 0.1^b^	10.7 ± 0.5^b^	10.3 ± 0.4^b^	11.5 ± 0.6^b^	9.7 ± 0.4^b^	10.3 ± 0.3^b^	10.7 ± 0.1^b^	28.3 ± 0.2^a^
Gram-negative bacteria										
*P. aeruginosa *	8.1 ± 0.3^b^	11.3 ± 0.3^a^	7.8 ± 0.2^b^	10.9 ± 0.2^a^	10.5 ± 0.3^a^	7.2 ± 0.5^b^	6.6 ± 0.4^b^	6.8 ± 0.7^b^	6.5 ± 0.6^b^	12.3 ± 0.6^a^
*K. oxytoca *	9.4 ± 0.2^c^	12.1 ± 0.5^b^	8.1 ± 0.2^cd^	11.9 ± 0.9^b^	11.4 ± 0.3^b^	8.2 ± 0.5^cd^	7.5 ± 0.5^d^	7.8 ± 0.1^cd^	7.9 ± 0.3^cd^	26.2 ± 0.5^a^
*P. vulgaris *	0.0 ± 0.0	0.0 ± 0.0	0.0 ± 0.0	0.0 ± 0.0	0.0 ± 0.0	0.0 ± 0.0	0.0 ± 0.0	0.0 ± 0.0	0.0 ± 0.0	23.3 ± 0.6
*S. enteritidis *	0.0 ± 0.0	0.0 ± 0.0	7.3 ± 0.2^b^	0.0 ± 0.0	0.0 ± 0.0	7.4 ± 0.3^b^	0.0 ± 0.0	0.0 ± 0.0	6.6 ± 0.5^b^	21.5 ± 0.2^a^
*E. coli *	10.3 ± 0.4^ab^	8.4 ± 0.2^b^	11.8 ± 0.3^a^	7.6 ± 0.6^b^	7.5 ± 0.5^a^	8.0 ± 0.2^b^	9.5 ± 0.1^ab^	7.6 ± 0.4^b^	7.5 ± 0.3^b^	11.3 ± 0.1^a^

Different superscript letters within the same row indicate significant differences (Tukey's test, *P* < 0.05) of the means for each solvent extract of the *Passiflora*. PE: petroleum ether; ACT: acetone; MET: methanol; STD: standard (chloramphenicol).
